# Prediction of meat spectral patterns based on optical properties and concentrations of the major constituents

**DOI:** 10.1002/fsn3.286

**Published:** 2015-09-23

**Authors:** Gamal ElMasry, Shigeki Nakauchi

**Affiliations:** ^1^ Department of Computer Science and EngineeringToyohashi University of Technology1‐1 Hibarigaoka TenpakuToyohashi441‐8580Japan; ^2^ Agricultural Engineering DepartmentFaculty of AgricultureSuez Canal UniversityIsmailiaEgypt

**Keywords:** Absorbance, Beer–Lambert, hyperspectral imaging, meat, optical properties, scattering, spectroscopy

## Abstract

A simulation method for approximating spectral signatures of minced meat samples was developed depending on concentrations and optical properties of the major chemical constituents. Minced beef samples of different compositions scanned on a near‐infrared spectroscopy and on a hyperspectral imaging system were examined. Chemical composition determined heuristically and optical properties collected from authenticated references were simulated to approximate samples' spectral signatures. In short‐wave infrared range, the resulting spectrum equals the sum of the absorption of three individual absorbers, that is, water, protein, and fat. By assuming homogeneous distributions of the main chromophores in the mince samples, the obtained absorption spectra are found to be a linear combination of the absorption spectra of the major chromophores present in the sample. Results revealed that developed models were good enough to derive spectral signatures of minced meat samples with a reasonable level of robustness of a high agreement index value more than 0.90 and ratio of performance to deviation more than 1.4.

## Introduction

There has been a resurgence of interest in developing objective methods for the simple and rapid analysis of raw materials and final products during quality assurance programs of meat and meat products. Being nominated for on‐line or at‐line implementations, optical method depending on interaction of light with the food samples has been one of the most successful techniques casted for food quality assessment to provide several quality details simultaneously (Fernández Pierna et al. 2012). The interaction of electromagnetic radiation with food samples in the form of reflection, transmission, and/or absorption depends profoundly on the physicochemical characteristics of these samples. The light‐sample interaction is dependent on the wavelength of the incident light, type of the sample and main chromophores in the sample (Hlavác 2013). Many experimental results showed significant correlations between the sample's biochemical composition and the corresponding reflectance or absorbance spectra recorded by spectral systems (Burger and Geladi 2006; ElMasry and Sun 2010; Kobayashi et al. 2012; Gowen 2014). The ability of light to penetrate a biological material, interrogate its components, and then escape the material for detection is the key to food evaluation applications. The short‐wave infrared light can penetrate relatively deep into biological soft materials due to the lower scattering property at NIR region than at visible region (Tsai et al. 2001). This makes NIR region a good spectral region for performing nondestructive measurements on thick or bulky biological samples (Wetzel 1983). The NIR absorption property of such samples varies with their constituents especially water, fat, protein, collagen, and their combination ratio.

Several analytical models capable of simulating light propagation in biological media and approximating various optical properties of biological samples have been developed. These models require a priori knowledge on the absorbing and scattering properties and allow for the calculation of the light distribution in the media. The measurement of light within or at the surface can also be used to determine sample's optical properties (van Veen 2006). Using the essential optical parameters of the main chromophores in the biological samples inevitably leads to development of robust models to predict basic optical and spectral properties (Allen et al. 1969; Baret et al. 1988; Yamada and Fujimura 1988). For instance, the model developed by Jacquemoud and Baret (1990) and its subsequent versions (Fourty et al. 1996; Jacquemoud et al. 1996; Baret and Fourty 1997; Bousquet et al. 2005; Feret et al. 2008) is one of the efficient models for simulating reflectance and transmittance over the whole optical domain with a minimum number of input optical parameters.

As the spectrum of a biological material in the visible and NIR regions results from the overtones and combinations of O–H, C–H, and N–H groups' stretching vibrations (Osborne et al. 1993; Ozaki et al. 2006; Prieto et al. 2009), spectral measurement is most likely to provide information about samples' composition, and this composition is correspondingly revealed by the spectral behavior of the sample. Given the fact that O–H, C–H, and N–H are the most abundant chemical bonds in biological samples, it postulated to develop a predicting method to approximate the basic spectral characteristics of a sample based on chemical constituents containing these bonds (i.e., water, fat, and protein as the major contributors at NIR spectral region). By assuming that the intrinsic absorption properties of each constituent do not change in spite of being alone or conjugated with other constituents, the absorption spectra could be approximated by knowing the optical properties of these constituents and their relative concentrations. The central hypothesis of this work is that the optical absorption parameters of water, fat, and protein are sufficiently well differentiated from those of the other constituents in the NIR spectral range to permit the detection of various spectral behaviors. This study aims to approximate absorption spectra of standardized minced meat samples with known concentrations of major constituents (water, protein, and fat). Rather than calculating optical properties for each constituent at every single wavelength separately, these data over the entire wavelength range were imported form relevant authenticated references. In this study, two basic approaches were tested based on the basic form of Beer–Lambert law: one without considering scattering effect and the other by using the model developed by Jacquemoud and Baret (1990) that considers scattering processes within the sample's internal structure. The later approach assumed that the sample is composed of a pile of elementary layers (*N*) separated by air spaces and each layer is characterized by a refraction index (*n*) and an absorption coefficient (*μ*_a_).

### Theoretical background

Chromophores are referred to the sample compounds which absorb light in the spectral region of interest. Each chromophore has its own particular absorption spectrum which describes the level of absorption at each wavelength (Branco 2007). The dominant chromophores in meat samples that absorb substantial electromagnetic spectrum in the NIR region (900–2500 nm) are moisture, protein, and fat with distinctive absorption features at certain wavelengths. In the visible range of the spectrum there are contributions from other dominant chromophores such as oxyhemoglobin (HbO_2_), deoxyhemoglobin (Hb), melanin, cytochrome c oxidase, myoglobin, etc. These chromophores absorb more light in the ultraviolet and visible region than the longer wavelengths regions. Therefore, these chromophores can be largely ignored in the NIR range, as they contribute little to the overall attenuation (Schmidt 2000). Optical properties of biological materials, particularly scattering and absorption coefficients and refractive index, quantitatively describe the light‐material interaction. In essence, a photon incident on a biological sample moves in all directions and may be scattered or absorbed and transmits its energy to the molecules causing excitation of the molecular electronic, vibrational, or rotational states (Hlavác 2013).

The simplest form of optical properties of biological tissues is the refractive index *n*, which determines the speed of light in a medium. Any change in the refractive index, either continuous or abrupt (at boundaries), gives rise to scattering, refraction, and reflection (Branco 2007). The refractive index *n* is a dimensionless number determining the speed of light c under the vacuum compared with its speed ν propagating within the sample tissue described by the following equation:

(1) n=c/v

At the interface between two media with different refractive index, refraction occurs and is described by Snell's law as:

(2) $$n_{1}\text{sin}\theta_{1}=n_{2}\text{sin}\theta_{2}$$

where *θ*_1_ is angle of incidence, and *θ*_2_ is angle of refraction. Basically, refractive index is related with the other essential optical properties because the complex refractive index, *n* = *n*′ + *jn*″, includes real and imaginary refractive indexes. The real index, *n*′*,* describes energy storage and hence affects the speed of light in a medium and determines the scattering properties of the sample tissue. If *n*′ values were dominantly constant at all wavelengths, it means that there would be no scattering. On the other hand, the imaginary refractive index, *n*″, describes energy dissipation and inevitably specifies the absorption coefficient, *μ*_a_ = 4*πn*″/*λ* (Jacques 2013). As sample tissues are heterogeneous in composition, one may need to know the refractive indexes for the various constituents or an averaged value for the sample as a whole. As the biological sample could be considered as turbid, inhomogeneous medium, it will have a refractive index higher than air (*n *=* *1) and it is usually considered to be around 1.4 for most biological specimens (Delpy et al. 1988).

Because the refractive index for any material is a function of wavelengths, it can be numerically approximated using different empirical equations in the NIR wavelength range (Ding et al. 2006).

(3) $$n\;=1.3696+\frac{3916.8}{\lambda^{2}}+\frac{2558.8}{\lambda^{4}}$$

To ensure that this equation gives accurate estimation of refractive indexes, values of refractive indexes calculated by this equation were compared with some reported values of refractive indexes of fresh meat muscles at certain wavelengths. There was a great coincidence between calculated and reported values in the visible and NIR ranges. For example, refractive index of porcine muscles was 1.38 and 1.37 at wavelengths of 632.8 and 1341.4 nm, respectively, as reported by Shuying et al. (2003) and Hui and Shusen (1996), which exactly the same values obtained from the abovementioned equation. Notwithstanding, the values of refractive index depend mostly on the characteristics of sample such as percentage of water, protein, fat, and other contents. More interestingly, there is no difference in refractive index between whole block of tissue and homogenized (mashed) tissues (Shuying et al. 2003).

On the other hand, the measured intensity *I* is dependent on the incident intensity *I*_o_, the light traveling path *l,* and the average absorption coefficient of the sample *μ*_a_ in an exponential manner:

(4) $$I=I_{o}e^{-\mu_{a}l}$$

where *μ*_a_ (cm^−1^) represents the probability of a photon's energy being absorbed by the molecules per unit length. Absorption coefficient describes how far photon can penetrate into sample tissue before being absorbed. Absorption can be also described by particle density *ρ* and absorption cross section *σ*_a_ (Hlavác 2013) as:

(5) $$\mu_{a}=\rho \sigma_{a}$$

The transmittance *T* is the ratio of transmitted intensity to the incident intensity (*I*/*I*_o_), and the absorbance *A* (representing the loss in light intensity), is related to the transmittance in the form:

(6) $$A\;=\;{\text{log}}_{10}\left(1/T\right)={\text{log}}_{10}\left(I_{o}/I\right)={\text{log}}_{10}\left(e^{\mu_{a}l}\right)$$

Combining previous equations leads to the following equation known as Beer–Lambert's law. As a fundamental theory for the measurement of chromophore concentration within a sample, this law relates the absorption of light to the properties of the sample through which the light is traveling.

(7) $$A=\mu_{a}l{\text{log}}_{10}e\;=0.4343\mu_{a}l=\varepsilon_{a}cl$$

where ε_a_ is the molar extinction coefficient or molar absorptivity in the unit of cm^−1^(mol/L)^−1^ (or cm^−1^ M^−1^), and *c* is the concentration of the absorber or chromophore inside the sample in the unit of mol/L (or M). Sometimes one wishes to describe the absorption properties of a material that does not have a well‐defined concentration, so that an alternative concentration must be used. For example, if concentration *c* was measured in unit (mg/mL), an alternative extinction coefficient must be used, *ε* (cm^−1^ [mg/mL]^−1^). The product *εc* still has units of cm^−1^, and *εcl* is dimensionless. So, while the literature usually uses *c* (M), *ε* (cm^−1^ M^−1^), and *l* (cm), alternative units for *c*,* ε,* and *l* may be used, as long as *εcl* is dimensionless (Jacques 2013). Alternative units such as cm^2^ mol^−1^ could be also used by substituting the concentration with the unit of mol/cm^3^. This change in units emphasizes the point that *ε* is a molar cross section for absorption analogous to the mass attenuation coefficient *μ*_a_/*ρ* (Singh et al. 2002). Generally, molar extinction coefficient depends upon the wavelength of the incident radiation and is greatest where the absorption is most intense. Equation (7) implies that the absorbance is linear with the concentration of chromophores. If the path length and the molar absorptivity are known and the absorbance is measured, the concentration of the substance can be deduced. The equation can be extended when the specimen contains several different absorbing chromophores. Thus, the absorption coefficient of a meat sample is the sum of contributions from all absorbing chromophores (water, protein, and fat) within the sample. Assuming homogeneous distribution of the major chemical compounds (chromophores) and because the detailed molecular composition of the sample is not well specified, the global or average absorption coefficient *μ*_a_ could be determined as:

(8) $$\mu_{a}=\sum f_{v.i}\mu_{a.i}$$

where *f*_v.*i*_ is the volume fraction (or mass fraction) of a chromophore *i* and the absorption coefficient of that pure chromophore is *μ*_a.*i*_. In case of a minced meat sample with three major chromophores (water, protein, and fat), the previous equation takes the following form:

(9) $$\mu_{a}=f_{w}\mu_{w}+f_{p}\mu_{p}+f_{f}\mu_{f}$$

where *f*_w_ *, f*_p_, and *f*_f_ are mass fraction of water, protein, and fat with corresponding absorption coefficients of *μ*_w_, *μ*_p_, and *μ*_f_, respectively.

In case of intact sample examined in the reflectance mode of spectroscopy or hyperspectral imaging systems, the absorption can be easily obtained using equation (7). However, in reflectance mode, the path length *l* can hardly be measured. The product *cl* in the previous equation (7) is thus replaced by *c*_eff_, named as effective concentration.

In the basic Beer–Lambert model shown in equation (6) or equation (7), it is assumed that the photon passes through the material without being scattered. In practice, photons are scattered into different paths inside inhomogeneous turbid materials like the meat sample, and some of them are simply lost after multiple scattering leaving only a small fraction of photons following the pathways and they are then collected by the detector. Photons will be scattered even in a very thin layer of the specimen before being absorbed. Due to the interaction with scattering particles, the direction of the photon is changed and after several numbers of scattering events light loses its initial trajectories. Scattering is mainly caused by changes in refractive index on the microscopic level. Scattering is also dependent on the wavelength and usually decreases with the increasing wavelength (Hlavác 2013). Obviously, scattering causes light to travel extra distance in the specimen, increasing the probability of photon absorption. Visible light will be greatly scattered and absorbed within the depth of just a few hundred microns. The relatively low absorptivity in the near‐infrared spectral region allow light to be detected after many scattering events in inhomogeneous materials (Pelletier and Pelletier 2010). This makes NIR region a good spectral region for performing nondestructive measurements on thick or bulky biological specimens (Wetzel 1983). Notwithstanding, many complicated mathematical models have successfully been developed to evaluate the absorption properties of different materials under absorption and scattering conditions. When Beer–Lambert law considered scattering process, the coefficient of scattering *μ*_s_ can be used instead of absorption coefficient *μ*_a_ in equation (6).

## Materials and Methods

### Datasets

Samples of minced beef with different concentrations of fat trimming were used as reference data (Morsy and Sun 2013). The dataset contains spectral and chemical constituent data of a series of minced beef samples with different chemical compositions. Each sample was first scanned in NIR spectroscopy in the reflectance mode followed by chemical composition assessment of water and fat contents using Smart Trac System (CEM Corporation, Matthews, NC) and protein content using LECO total nitrogen determinator (Model FP‐428; LECO R Corporation, St. Joseph, MI). Mean reflectance spectrum in both visible and NIR range 400–2498 nm was collected for each sample using NIR system (Model 6500; Foss NIRSystems Inc., Laurel, MD) with 14 nm incremental interval yielding 150 data points per spectrum. However, the spectral data were adapted to 5 nm incremental interval with 421 data points using an interpolation algorithm. The absorbance was then calculated as (Absorbance = log [1/Reflectance]) for subsequent modeling. As some important chromophores such as hemoglobin and melanin concentrated in the visible range of the spectrum were unavailable in this dataset, only the data in the NIR range of 900–2400 nm were used for testing the models. The obtained dataset represents a wide range of internal structure and chemical composition of different concentrations of water, protein, and fat contents. The spectral patterns of these samples were approximated by the proposed models and then compared with those reported by the reference.

As our task is to confirm the capability of the models in predicting spectral patterns despite the source of acquisition devices, another dataset of other minced meat samples scanned in a NIR hyperspectral imaging system in the spectral range 900–1700 nm as described by ElMasry et al. (2013) were also tested. The acquired imaged were standardized by extra two reference images to calculate absorbance spectra at each single pixel in the image. The basic concept of hyperspectral imaging originated from the fact that the amount of radiation that is reflected, absorbed, or emitted varies with wavelength and conveyed to each single pixel in the image. Average spectra were then extracted from each image to represent the whole sample being used for the comparison with the approximated spectra calculated from the prediction models explained below. As the extracted spectral data from hyperspectral images were very noisy at both edges of the spectrum, only spectral data in the spectral range 950–1650 nm were considered.

Digital values registered by the camera are limited by its dynamic range and the number of bytes assigned to each pixel to represent the light intensity. Dynamic range represents the camera's ability to display/reproduce the brightest and darkest portions of the image and how many variations in between. The largest possible signal is directly proportional to the maximum number of electrons per pixel (i.e., the full well capacity of the pixel). In other words, it describes the ratio between the maximum and minimum measurable light intensities from the sample. Dynamic range is not equal to digitization level such that a camera with a 12‐bit analog/digital (A/D) converter does not necessarily have 12 bits of dynamic range because this does not consider the noise that contains sensor readout noise and dark current noise. For simplicity, during this context of this study we considered the DV (digital values) only without going through more sophisticated details in the optical and hardware complexity. For example, camera used in the hyperspectral system was of a 12‐bit A/D converter, which provides digital count values in the range 0–4095.

For quantitative assessment, it is preferable to convert the raw DV of the hyperspectral image data to physical quantities before using the data to interpret the composition of the sample. Important physical quantities include radiance, reflectance, or transmittance. As an image is a resultant of both illumination of the light source and reflected radiance from the sample, the DV registered by the camera could be converted to intensity values at any wavelength for any pixel in the image using the following formula

(10) $$I_{\lambda }=a.\text{DV}+b$$

where *a* and *b* are the gain and offset for each spectral channel (wavelength). Also, it is well‐known that the key relationship between the illuminating/incident irradiance (*F*_λ_) and the reflected radiance (*I*_λ_) is given by the following formula using spectral reflectance (*R*_λ_):

(11) $$I_{\lambda }=R_{\lambda }F_{\lambda }/\pi$$

Also, it is important to emphasize that the total reflected radiance received by the detector (*I*_All_) is a sum of the upward reflected radiance from the sample (*I*_Sample_) and any accompanying or complementary radiance (*I*_Comp_) resulting from the surroundings or even from the camera dark current.

(12) $${\left(I_{\mathrm{All}}\right)}_{\lambda }={\left(I_{\mathrm{Sample}}\right)}_{\lambda }+{\left(I_{\mathrm{Comp}}\right)}_{\lambda }$$

The complementary radiance depends on the strength of the illumination and the density of scattering particles in the field of view. It will be a decreasing function of wavelength, because shorter waves are scattered more than long waves as stated before (Tsai et al. 2001; Hlavác 2013). Owing to this trend, it can often be ignored in the NIR (near‐infrared). Therefore, in case of a typical dark object with reflected radiance equaling zero, the total radiance of the detector will exhibit the commentary radiance. In general, the reflected radiance of the sample will equal what is read in the camera detector minus the complementary radiance.

(13) $${\left(I_{\mathrm{Sample}}\right)}_{\lambda }={\left(I_{\mathrm{All}}\right)}_{\lambda }-{\left(I_{\mathrm{Comp}}\right)}_{\lambda }$$

Applying equations (10), (11), and (13) on standard white and black surfaces with maximum ‘one' and minimum ‘zero' reflectance value, respectively, at each wavelengths results in:

(14) $$R_{D}=\frac{\pi }{F_{\lambda }}\left[a.{\text{DV}}_{D}+b-I_{\mathrm{Comp}}\right]=0$$

(15) $$R_{W}=\frac{\pi }{F_{\lambda }}\left[a.{\text{DV}}_{W}+b-I_{\mathrm{Comp}}\right]=1$$

(16) $$R_{\mathrm{Sample}}=\frac{\pi }{F_{\lambda }}\left[a.{\text{DV}}_{\mathrm{Sample}}+b-I_{\mathrm{Comp}}\right]$$

Equation (14) leads to *b* − *I*_Comp_ = ‐ *a*.DV_D_

Subtracting equation (14) from equation (15) leads to

(17) $$R_{W}-R_{D}=\frac{\pi }{F_{\lambda }}\left[a.{\text{DV}}_{W}+b-I_{\mathrm{Comp}}\right]-\frac{\pi }{F_{\lambda }}\left[a.{\text{DV}}_{D}+b-I_{\mathrm{Comp}}\right]=1$$

(18) $$\frac{F_{\lambda }}{\pi }=a\left({\text{DV}}_{W}-{\text{DV}}_{D}\right)$$

Substituting both (17) and (18) in (16) leads to the well‐known equation of calculating reflectance of the sample using the DV recorded by the camera for the sample as well as for white and dark references.

(19) $$R_{\mathrm{Sample}}=\frac{{\text{DV}}_{\mathrm{Sample}}-{\text{DV}}_{D}}{{\text{DV}}_{W}-{\text{DV}}_{D}}$$

The resulting reflectance (*R*_Sample_) values could be converted easily to absorbance (*A*_Sample_) values called virtual absorbance or pseudoabsorbance to comply with the Beer–Lambert's law using the following expression:

(20) $$A_{\mathrm{Sample}}=\text{log}\;\left(1/R_{\mathrm{Sample}}\right)$$

### Data modeling

#### Model 1

Although it is a smart method to find a sample's chemical composition nondestructively and without specific sample preparation, the conventional spectroscopy is unfortunately unable to provide composition gradients and to provide information about the heterogeneity of the samples being analyzed because it has virtually no spatial information and it only collects the aggregate amount of light reflected, emitted or transmitted from a small area of a sample (a single‐point measurement where the sensor is located). Accordingly, the approximation models assume a homogeneous distribution of the major constituents in the tested samples. The first method for approximating spectral signature of a minced meat sample depends on using the basic form of Beer–Lambert's law (Equations (6) and (7)) ignoring scattering process to calculate the absorbance values at each wavelength in the NIR range. The measured concentrations (or mass fraction) of water, protein, and fat contents and their corresponding absorption coefficients collected from relevant references were used for calculations. By assuming a constant path length, substitution of constituents' given data (absorption coefficients and mass fractions) directly into equation (9) *μ*_a_ = *f*_w_ *μ*_w_ + *f*_p_ *μ*_p_ + *f*_f_ *μ*_f_ and equation (7) *A*_Sample_ = 0.4343*μ*_a_ *l* gives an approximation of the average absorbance (*A*) of the examined sample. This can be written in a matrix form as:

(21) $$\left[\begin{array}{c} A_{\lambda 1}\\ A_{\lambda 2}\\ A_{\lambda 3}\\ .\\ .\\ A_{\lambda n} \end{array}\right]={\text{log}}_{10}\left(e\right)\cdot \overset{A_{\mathrm{Sample}}=0.4343\mu_{a}l}{\overbrace{\left[\begin{array}{ccc} \mu_{W\lambda 1} & \mu_{P\lambda 1} & \mu_{F\lambda 1}\\ \mu_{W\lambda 2} & \mu_{P\lambda 2} & \mu_{F\lambda 2}\\ \mu_{W\lambda 3} & \mu_{P\lambda 3} & \mu_{F\lambda 3}\\ . & . & .\\ . & . & .\\ \mu_{W\lambda n} & \mu_{P\lambda n} & \mu_{F\lambda n} \end{array}\right]\cdot \left[\begin{array}{c} F_{W}\\ F_{P}\\ F_{F} \end{array}\right]}}\cdot l$$

#### Model 2

The method explained by Jacquemoud and Baret (1990) was used to model the optical parameters of the major constituents in the sample and their corresponding concentrations to reconstruct the reflectance values at every wavelength using a structural parameter (*N*) that mimics the scattering process. In this model, the fundamental optical characteristics such as absorption and scattering were exploited. As the analyzed samples were minced meat, it is assumed that the samples have a pile of *N* homogeneous layers separated by *N*−1 air spaces as the infinite reflectance is a simple function of the ratio between the absorption and the scattering coefficients. Scattering is described by the refractive index of sample materials (*n*) and *N* parameter. As water is the main component of the meat sample the refractive index was used in the model. The model based on the “plate model” developed by Allen et al. (1969) and its modified form that assumes that when a light ray penetrates inside a layer of a biological object, the light flux is assumed to be diffuse and isotropic. Then the reflectance *ρ*_90_ and transmittance *τ*_90_ in the elementary layer can be written as follows for a given wavelength:

(22) $$\rho_{90}=\left(\rho_{\alpha }-y\right)/x$$

(23) $$\tau_{90}=\tau_{\alpha }/x$$

where

(24) $$\rho_{\alpha }=\left[1-t_{\mathrm{av}}\left(\alpha ,n\right)\right]+\frac{t_{\mathrm{av}}\left(90,n\right)t_{\mathrm{av}}\left(\alpha ,n\right)\theta^{2}\left[n^{2}-t_{\mathrm{av}}\left(90,n\right)\right]}{n^{4}-\theta^{2}{\left[n^{2}-t_{\mathrm{av}}\left(90,n\right)\right]}^{2}}$$

(25) $$\tau_{\alpha }=\frac{t_{\mathrm{av}}\left(90,n\right)t_{\mathrm{av}}\left(\alpha ,n\right)\theta n^{2}}{n^{4}-\theta^{2}{\left[n^{2}-t_{\mathrm{av}}\left(90,n\right)\right]}^{2}}$$

(26) $$x\;=\frac{t_{\mathrm{av}}\left(\alpha ,n\right)}{t_{\mathrm{av}}\left(90,n\right)}$$

and

(27) $$y=1-\frac{t_{\mathrm{av}}\left(\alpha ,n\right)}{t_{\mathrm{av}}\left(90,n\right)}$$

*t*_av_(α, *n*) is the transmissivity of a plane surface averaged over all directions of incidence and over all polarizations. It is a rather complex expression but it can be exactly calculated based on the refractive index *n* values at each single wavelength, transmission coefficient *θ* and incident angle α. The transmission coefficient *θ* at each single wavelength is related to the absorption coefficient through the following expression (Allen et al. 1969):

(28) $$\theta -\left(1-\mu_{a}\right)e^{-\mu_{a}}-\mu_{a}^{2}\int_{\mu }^{\infty }x^{-1}e^{-x}dx=0$$

The integration part of the previous equation could be put in the form $\int_{\mu }^{\infty }\frac{e^{-x}}{x}dx$ which represents the typical form of the exponential integral that can be easily solved using the Matlab function “expint” written as ‘expint (*μ*_a_)'. Hence, the average absorption coefficient *μ*_a_ was first calculated form equation (9) *μ*_a_ = *f*_w_ *μ*_w_ + *f*_p_ *μ*_p_ + *f*_f_ *μ*_f_ by using the mass fractions of water, protein, and fat and their corresponding absorption coefficients from relevant literatures Kou et al. (1993), Buiteveld et al. (1994), Jacquemoud et al. (1996), Altshuler et al. (2003) and van Veen et al. (2004), respectively. The resulting value of absorption coefficient *μ*_a_ was then used in equation (28) to calculate the transmission coefficient *θ* that correspondingly used to calculate reflectance and transmittance at an incident angle α using equations (24) and (25), respectively. Hence, the ‘total' reflectance and transmittance for *N* layers are given by the following equation:

(29) $$R_{N,\alpha }\;=xR_{N,90}+y$$

and

(30) $$T_{N,\alpha }=xT_{N,90}$$

where the total reflectance and transmittance in the normal direction (*R*_*N*,90_ and *T*_*N*,90_) are estimated using the following equation according to Stock's homogeneous system:

(31) $$\frac{R_{N,90}}{b_{90}^{N}-b_{90}^{-N}}=\frac{T_{N,90}}{a_{90}^{}-a_{90}^{-1}}=\frac{1}{a_{90}b_{90}^{N}-a_{90}^{-1}b_{90}^{-N}}$$

where

(32) $$a_{90}=\left(1+\rho_{90}^{2}-\tau_{90}^{2}+\delta_{90}\right)/2\rho_{90}$$

(33) $$b_{90}=\left(1-\rho_{90}^{2}+\tau_{90}^{2}+\delta_{90}\right)/2\tau_{90}$$

(34) $$\delta_{90}=\sqrt{{\left(\tau_{90}^{2}-\rho_{90}^{2}-1\right)}^{2}-4\rho_{90}^{2}}$$

In this study, the calculations assumes that the incident angle α was typically 45°, number of layers *N* was assumed to equal 25 and the refractive index values at different wavelengths in the NIR range were calculated from equation (3). Running the model under these assumptions was expected to give a reasonable estimation of the average reflectance values of the tested samples at different wavelengths. The estimated reflectance was then converted to absorbance values using equation (20) *A*_Sample_ = log (1/*R*_Sample_) being compared with the actual measured absorbance values of the samples to check the approximation accuracy of the models.

## Results and Discussion

### Optical properties and spectral features

**Figure 1:**
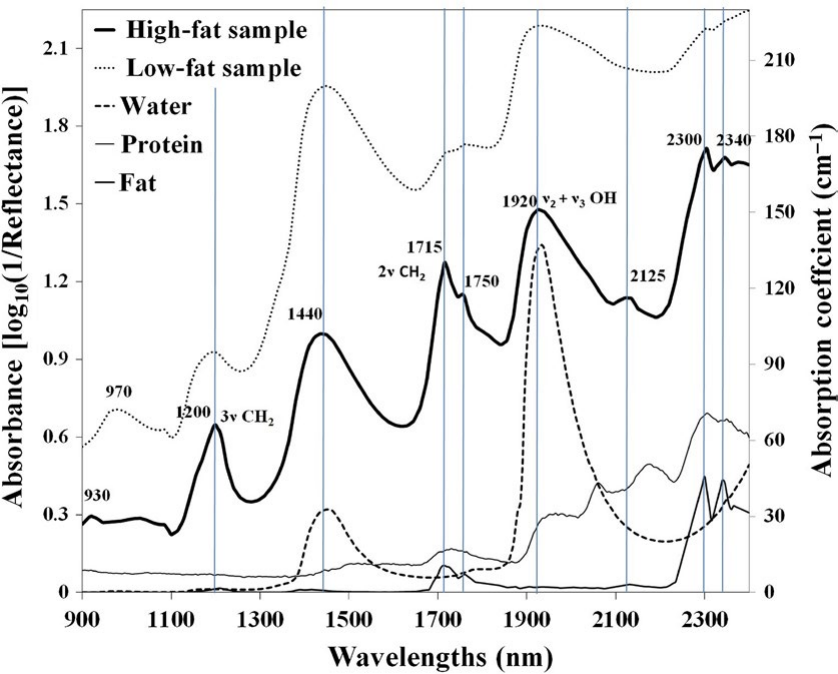
(right axis) Absorption coefficients of water (Kou et al. 1993; Buiteveld et al. 1994), protein (Jacquemoud et al. 1996), and fat (Altshuler et al. 2003; van Veen et al. 2004) in the near‐infrared spectral range 900–2400 nm, (left axis) spectral signature of low‐ and high‐fat content samples.

The absorption coefficient of pure water, protein, and fat as the major chromophores in meat samples in the NIR range are shown in Figure 1. Also, the figure displays the measured spectral absorption of two different samples: one of high‐fat content (80.60%) and the other of low‐fat content (2.49%) to demonstrate the remarkable absorption peaks compared with the basic chromophores. In general, the corresponding absorption coefficients curves of the main components display classical features with little spectral shifts of the principal absorption peaks compared to those observed in the real meat samples. As the figure demonstrates the absorbance in the NIR range only, it is expected not to see absorption peaks of other chromophores such as hemoglobin (oxygenated and/or deoxygenated forms) and melanin as they contribute little to the overall attenuation (Schmidt 2000). The absorption properties of hemoglobin and its derivatives are lower than that of water and fat in the wavelength region beyond 1000 nm. Thus, NIR infrared absorption of these chromophores was rather weak in long wavelength region (Tsai et al. 2001), and the propagation of light becomes diffuse (Niemz 2007). From Figure 1, it can be seen that water (dashed line) has low absorption coefficients at the beginning of the NIR range and then exhibits remarkable peaks at 1440 and 1920 nm. In fact, samples containing a certain chromophore should exhibit its absorption peaks in spite of the spectral shift. Accordingly, the same two peaks of water were explicitly recognized in the measured spectra of mince samples with high absorbance values of low‐fat content sample as it contains high content of water. As reported in literatures (Xu et al. 2010; Wang et al. 2013), water has absorption peak at 970 nm, where the high‐order combination bands of symmetric and asymmetric stretching modes of the O–H bond reside. However, this peak was rather difficult to discern due to axis scale and its magnitude is smaller than that at longer wavelengths (1440 and 1920 nm).

Similarly, the absorption peaks of fat appeared in the absorption coefficient curve of pure fat at 1200, 1715, 1750, 2125, 2300, and 2340 nm were correspondingly noticeable in the sample spectra especially in the high‐fat content sample. In Figure 1, the two small absorption peaks at 930 and 1040 nm in the high‐fat content spectrum are the combinations of stretching and bending of the methyl and methylene (CH_2_) groups in fatty acids (Ozanich et al. 1992). The large absorption peak near 1200 nm is the second overtone of the C–H stretching vibration in fatty acids. Absorption bands 1715 and 1750 nm related to C–H first stretching overtones and at 2125, 2300, and 2340 nm related to C–H combination tones (Cozzolino et al. 2002; Hoving‐Bolink et al. 2005) are all characteristics of fat and fatty acid molecules in the sample. Nevertheless, two ‘valleys' exist in the water absorption curve at 1000–1300 and 1600–1850 nm. Importantly, the second and first overtone transitions of C–H bonds are located at these two spectral windows, respectively. These spectral features produce two optical windows for C–H bond‐selective imaging (Xu et al. 2010; Wang et al. 2013).

On the other hand, as the volume fraction of proteins in the sample was relatively small and probably <5% in high‐fat content samples, the contribution by protein absorption peak in minced samples was very small and buried in the strong absorption peak of water and fatty acids at the same wavelength regions (Tsai et al. 2001) because it is responsible for binding most of water content in meat samples. The difference among samples in protein content related to N–H overtones was usually observed at 1187, 1510, 1690, and 2265 nm (Park et al. 2001), but most of them were rather difficult to be discerned from the sample spectra due to their closeness to fat and water absorption bands as these bands are very broad (Morsy and Sun 2013). As the position of the fundamental absorption bands in the NIR region is very well documented, they can be used as a starting point in the prediction of the corresponding overtone bands seen in the NIR spectrum.

### Approximation of spectral patterns

**Figure 2:**
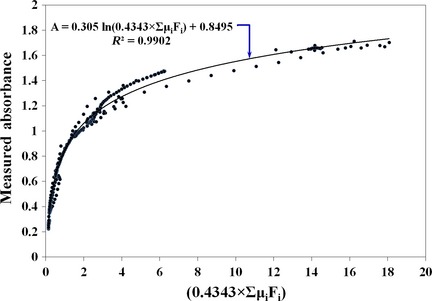
Development of a relationship to calculate absorbance value ‘*A*' from absorption coefficients *μ*_a_ = 0.4343 ∑ *μ*_a.*i*_ *f*_v.*i*_ in the near‐infrared spectral range.

In model 1, without considering the path length value, equation (9) calculates only the overall absorption coefficient *μ*_a_ = 0.4343 ∑ *μ*_a.*i*_ *f*_*i*_ and it does not give an estimation of the absorption itself. Because path length *l* values are unavailable of the examined minced meat specimens, the calculated overall absorption *μ*_a_ coefficients are plotted against the measured values of absorption spectrum of only one sample. The resulting exponential relationship was then used to convert the calculated absorption coefficient *μ*_a_ to absorbance *A* as shown in Figure 2.

**Figure 3:**
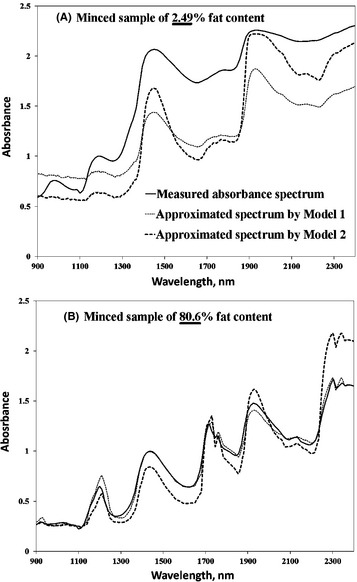
Approximated spectra resulted from Model 1 (dotted line) and Model 2 (dashed line) for minced meat samples of (A) low‐fat content (2.49%) and (B) high‐fat content (80.6%).

Figure 3 shows spectra produced by both models (Model 1 and Model 2) for samples of very low (2.49%) and very high (80.60%) fat contents. It was very astonishing to find spectral peaks and valleys of the approximated spectra by both models were positioned at the same locations as those of the original measured spectra. However, both models failed to locate the O–H absorption peak at 970 nm because the magnitude of the absorption coefficient value at this particular band was rather low compared to the other absorption peaks of water at 1440 and 1920 nm as stated before. Notwithstanding, the accuracy of approximating spectra in case of samples of low‐fat content was not so high, but it was generally concurred with the spectral patterns of the measured sample. This could be attributed to the physical properties of those samples such as particle sizes, particle size distribution, and bulk and/or compact density. To overcome this problem, various spectral transformations could be tried to suppress the physical information in NIR spectra because it obscures the chemical information (concentrations of the chromophores). The accuracy was augmented in case of samples of high‐fat content in which both models succeeded to identify all CH absorption peaks at 930, 1200, 1715, 1750, 2125, 2300, and 2340 nm precisely with almost the same values of the absorption magnitudes as the original measured spectra.

**Figure 4:**
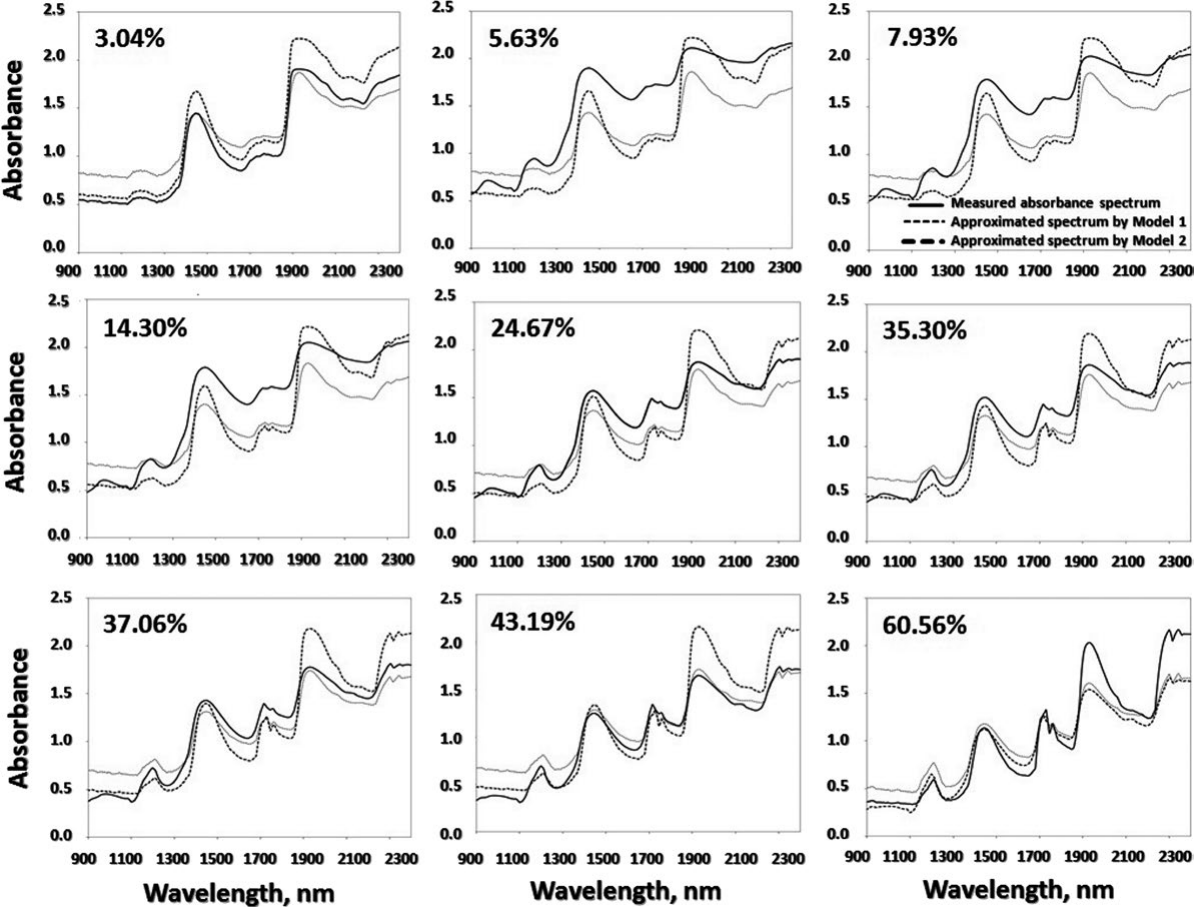
Comparison between measured and approximated spectra produced by Model 1 (dotted line) and Model 2 (dashed line) for minced meat samples of different fat contents (3.04–60.56%). The measured spectra (solid line) are extracted from minced samples scanned by near‐infrared spectroscopy.

**Figure 5:**
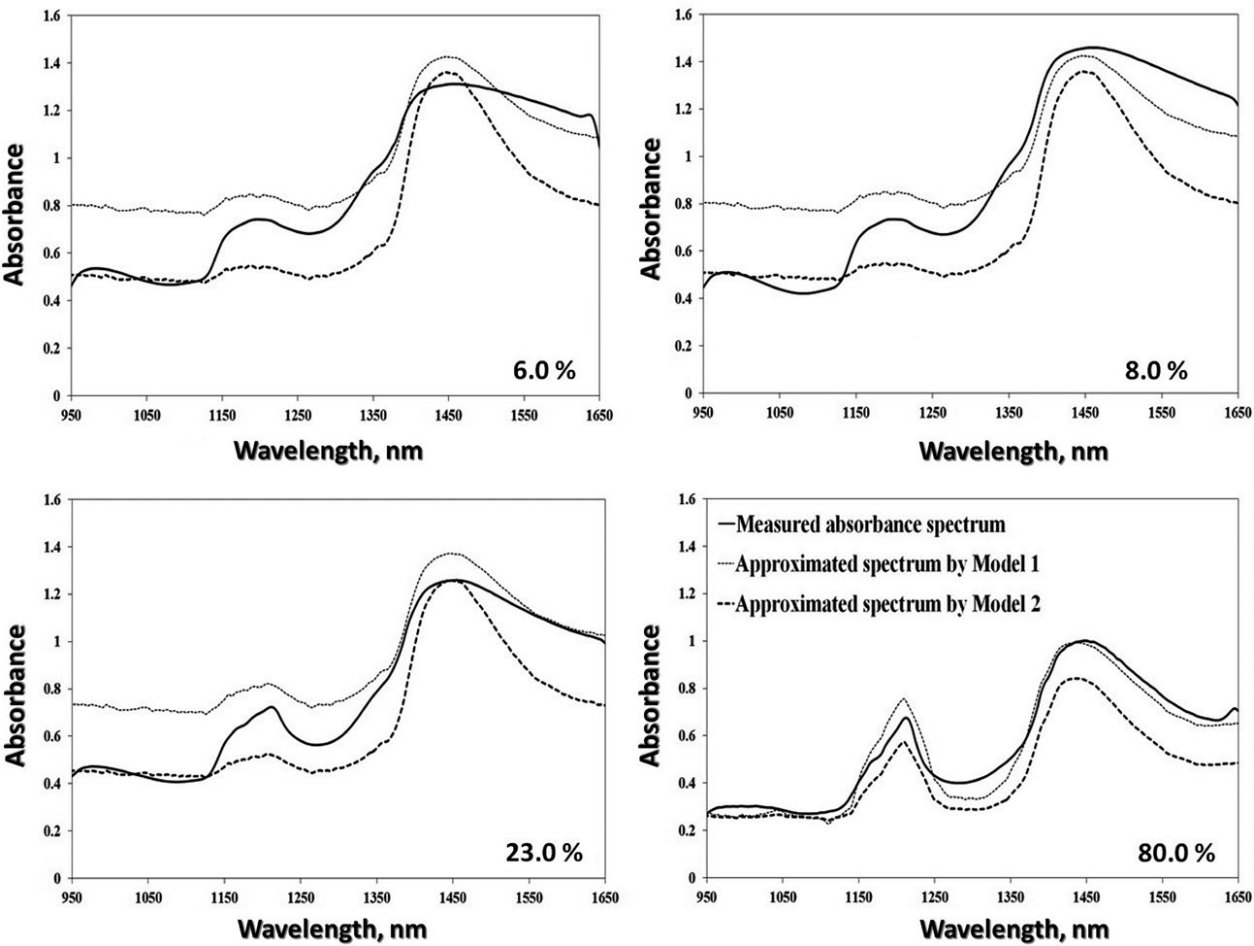
Comparison between measured and approximated spectra produced by Model 1 (dotted line) and Model 2 (dashed line) for minced meat samples of different fat contents (6.0–80.0%). The measured spectra (solid line) are extracted from minced samples scanned by near‐infrared hyperspectral imaging system.

Similarly, the resulting approximated spectra from both models compared with the original measured spectra of samples of different chemical compositions are shown in Figures 4 and 5 for spectral data collected using NIR spectroscopy and from hyperspectral images, respectively. As a general attitude, spectral behavior was found to be similar to that of the measured spectral signature for each sample. More positively, the smoothed spectral curves obtained implied that the illustrated spectra could be used to estimate spectral responses of the samples for any wavelength of interest in the examined NIR range. This is not applicable for the visible region where there is a trend for the approximated data to underestimate or overestimate the measured spectral values because some essential components such as hemoglobin and melanin that are dominant in the visible range (van Veen 2006) have not been considered in this study. Actually, there was some variation between approximated and original spectra that could be attributed to profound effects of some vital factors such as: (1) difference in environmental conditions such as humidity and temperature at which reference optical properties were measured and those at which spectral data extracted, (2) inconsideration of other chemical components (chromophores) such as gelatin, collagen, fiber, etc., or any other minor chromophores (3) unavailability of experimentally refractive index values that should be estimated for the examined samples, and (4) unavailability of absorption coefficients for all components under experimental conditions. Moreover, if the real values of path length were measured heuristically or the extinction coefficients (*ε*) were available for the chromophores in consideration, the performance of the model could be positively improved in approximating the spectral signatures. Indeed, if one is interested in spectroscopic detection, then the minor contributions are important, but if one is interested in understanding light penetration into the sample and its behavior after penetration, then the minor contributions usually do not significantly perturb the light transport (Jacques 2013).

### Model evaluation

Variation in performance of spectral prediction for samples of different chemical compositions by the two proposed models should be evaluated with some objectively statistical measures. Therefore, the approximation capacity of the models was evaluated by calculating RMSE (root‐mean‐square error), the RPD (ratio of performance to deviation), and index of agreement *d*. The RMSE calculated from the following equation is a measure of the differences between spectral values predicted by a model ${\hat{y}}_{k}$ and the spectral values actually measured *y*_*k*_ at any wavelength *k*. The value of RMSE represents the standard deviation of ‘residuals' or the differences between predicted values (by the model) and measured values to aggregate the magnitudes of the errors throughout the whole spectrum into a single parameter as a measure of accuracy.

(35) $$\text{RMSE}=\sqrt{\frac{\sum_{k=1}^{n}{\left({\hat{y}}_{k}-y_{k}\right)}^{2}}{n}}$$

On the other hand, the RPD is a capacity parameter that is defined as the relationship between the SD (standard deviation) of the original measured spectrum and the SE (standard error) (or ‘RMSE') of the approximated spectrum by the model (Williams 2003; González‐Martín et al. 2007). The higher the value of RPD was, the greater the probability of the model to approximate the spectra would be. According to the value of RPD, three categories could be identified to indicate the accuracy of approximating the spectrum. The RPD value >2.0 denotes robust models that can accurately predict the spectrum, models of RPD values in the range 1.4–2.0 are intermediate models that can be possibly improved, and models falling in RPD values <1.4 are unreliable that have no prediction ability (Chang and Laird 2002).

Moreover, index of agreement *d* suggested by Willmott (1982) and used by some other authors (Willmott et al. 1985; Sivacoumar and Thanasekaran 2001) was calculated by the following equation for the assessment of models' accuracy. The mean value of the measured and predicted spectrum indicates the position of central value about which the measurements are distributed, and these two should be as close as possible for a good model.

(36) $$d=1-\frac{\sum_{k=1}^{n}{\left({\hat{y}}_{k}-y_{k}\right)}^{2}}{\sum_{k=1}^{n}{\left(\left|{\hat{y}}_{k}-\overset{¯}{y}\right|+\left|y_{k}-\overset{¯}{y}\right|\right)}^{2}},{}_{}^{}{}_{}^{}0\leq d\leq 1$$

The calculated values of these three statistical parameters (RMSE, RPD, and *d*) are presented in Table 1. Basically, the ideal model should have value of *d* close to 1, RMSE close to zero and RPD >2. By deep contemplation of the results shown in Table 1, one can figure out that the capacity of spectrum approximation by both models was reasonably adequate for determining spectral signatures of minced meat samples of different fat contents. For spectral data either from spectroscopy or hyperspectral images, the quantitative statistical measures recommended above concur with the more qualitative, graphic representations illustrated in Figures 3, 4, 5. Except the sample of very low‐fat content, the values of RPD of both models in all cases was >1.4 with RMSEs ranging from 0.043 to 0.402 indicating robust or intermediate models that can be possibly improved.

**Table 1:** Statistical measures to evaluate models' ability to approximate the spectral signatures of minced meat samples having a wide range of fat contents

Fat content (%)	Model 1			Model 2		
	RMSE	RPD	*d*	RMSE	RPD	*d*
Spectra from NIR spectroscopy						
2.49	0.525	1.105	0.765	0.453	1.282	0.867
3.04	0.198	2.540	0.946	0.186	2.704	0.972
5.63	0.402	1.432	0.826	0.353	1.517	0.907
7.93	0.311	1.676	0.882	0.276	1.888	0.940
14.30	0.322	1.680	0.879	0.275	1.971	0.942
24.67	0.183	2.647	0.951	0.219	2.215	0.959
35.30	0.174	2.834	0.959	0.202	2.441	0.965
37.06	0.144	3.307	0.970	0.197	2.418	0.965
43.19	0.139	3.251	0.971	0.220	2.061	0.955
60.56	0.212	2.745	0.952	0.210	2.775	0.957
80.60	0.043	6.144	0.998	0.182	2.403	0.966
Spectra from NIR hyperspectral images						
6.0	0.167	1.916	0.924	0.202	1.582	0.914
8.0	0.198	1.959	0.904	0.251	1.549	0.888
23.0	0.170	1.802	0.912	0.161	1.907	0.933
80.0	0.046	5.155	0.992	0.139	1.716	0.919

RMSE, root‐mean‐square error; RPD, ratio of performance to deviation; NIR, near‐infrared.

Closeness to the original measured spectrum was evaluated using the values of agreement index (*d*) that can tell which model is superior over another in approximation. The values of *d* indicated that both models performed very well in approximating sample spectrum, but Model 2 was slightly more accurate when fat content was <24.67%. Above this threshold, both models performed almost the same in approximation with *d* values over 0.95. Consistent with the patterns of *d* values shown in Table 1, the magnitudes of RMSE errors indicate that Model 2 produces relatively smaller errors and larger values of RPD (> 2.0) at or above this threshold. The RPD value above 2.0 for both models confirms a very good potential of prediction for these two models.

Indeed, by accurate estimation of sample's spectral signature by either model, the optical properties of each chromophore could be then predicted with acceptable accuracy if the difference between the original and approximated spectra was mathematically minimized. This of course could be modeled in a retrieval process to estimate the optical properties of the chromophores concomitant with their corresponding concentrations. This step is out of the scope of the current paper but should be considered in our future research endeavors in more details.

## Conclusion

In the near‐infrared domain, the absorption features resulting from the combination of harmonics and overtones of the fundamental frequencies of each chemical bond was used as the input to approximate the spectral patterns of minced meat samples of different chemical compositions. Two datasets of samples were examined from the literature, the first were spectral data in the NIR of 900–2400 nm for samples scanned by NIR spectroscopy and the second were spectral data in the range 950–1650 nm from samples scanned by NIR hyperspectral imaging system. The theoretical background of the optical properties was first elaborated followed by testing two different models. Models exploited the optical properties of the main chromophores in the meat samples and their corresponding concentration for approximating samples' spectral signatures. The models' performance was evaluated comprehensively and objectively by some statistical parameters to compare the original signatures of the samples with those computed by both models. Results revealed that developed models were accurate enough to derive spectral signatures of minced meat samples with a reasonable level of robustness and consistent with prevailing scientific theory. Possible alternatives of improving the capability of the simulation model should be introduced as the model's performance changes with the chemical composition of the samples by considering particle size, particle size distribution and compactness of the samples. However, the proposed model is very useful to give an overview of the spectral behavior of the examined samples as an expeditious way of quality evaluation. Indeed, when applied to intact samples, retrieval of the chemical composition is much more complex due to the strong absorption of water that masks the weakest absorption features of some major compounds such as protein and other minor components. Also, sample structure adds another tough burden of the problem and represents additionally confounding factors that complicate the assessment of the optical properties. More importantly, the proposed models could have relevant impacts on industrial applications for food industry that needs to know how their products will look like in the market under different storage conditions that alter products' chemical composition.

## Conflict of Interest

None declared.
